# The Dual Role of Nitric Oxide in Guard Cells: Promoting and Attenuating the ABA and Phospholipid-Derived Signals Leading to the Stomatal Closure

**DOI:** 10.3389/fpls.2016.00476

**Published:** 2016-04-14

**Authors:** Ana M. Laxalt, Carlos García-Mata, Lorenzo Lamattina

**Affiliations:** Molecular and Integrative Physiology, Instituto de Investigaciones Biológicas, CONICET-Universidad Nacional de Mar del PlataMar del Plata, Argentina

**Keywords:** guard cells, stomata closure, abscisic acid (ABA), nitric oxide, phospholipid-derived signals, attenuation of hormone-induced signaling

## Overview of the ABA-induced signaling leading to the regulation of stomatal movement

Plants regulate the gas exchange with the environment through microscopic pores formed by specialized cells called guard cells that constitute the stomata. The control of water loss and CO_2_ uptake of plants relies on the size of the stomatal pore. Abscisic acid (ABA) is the master hormone governing the intricate network of molecular switches and physiological responses of guard cells that determine the degree of stomatal aperture. Once plants sense water deficit, ABA is synthesized, and enters the guard cells triggering a series of signals that result in stomatal closure and preservation of the water status of the whole plant. ABA signaling in guard cells involves several mechanisms sustained by enzymes, small molecules, and second messengers that finally promote the inactivation of inward-rectifying K^+^ (I_K, in_) channels, activation of outward-rectifying K^+^ (I_K, out_) channel, and activation of slow and rapid-anion channels (MacRobbie, 2006), resulting in the facilitation of solute efflux from guard cells and stomatal closure. The ABA receptor is a complex structure formed by a family of soluble proteins known as pyrabactin resistance/regulatory component of ABA receptor (PYR/PYL/RCAR) (Ma et al., 2009; Park et al., 2009), which interacts with a protein phosphatase-kinase complex, functioning as a double negative regulatory system (Umezawa et al., 2009; Vlad et al., 2009). The phosphatases ABA insensitive 1 (ABI1), ABA insensitive 2 (ABI2), and homology to ABI1 (HAB1) belong to clade A type 2C protein phosphatase (PP2C) and the kinases belong to the group III of the sucrose non-fermenting 1 (SNF1)-related protein kinase 2 SnRK2.2; 2.3; and the 2.6, the last one also known as open-stomata 1 (OST1) (Kulik et al., 2011). Once ABA binds to its receptor, it generates a conformational change of the PYR/PYL/RCAR-ABA complex that promotes the binding of PP2C allowing the phosphorylation, and hence the activation, of SnRK2. Downstream, SnRK2 phosphorylates numerous target proteins involved in ABA responses, including the NADPH oxidase (NADPHox) respiratory burst oxidase homolog F (RbohF) (Sirichandra et al., 2009). Plant NADPHox RbohD and RbohF play an active role in the production of reactive oxygen species (ROS) during ABA-induction of stomatal closure. Furthermore, it has been recently found that activated OST1 interacts with type 2A protein phosphatase (PP2A)-subunits (Waadt et al., 2015), which are functional proteins proposed to positively and negatively regulate the ABA signaling in guard cells (Kwak et al., 2002; Pernas et al., 2007).

**Figure 1:**
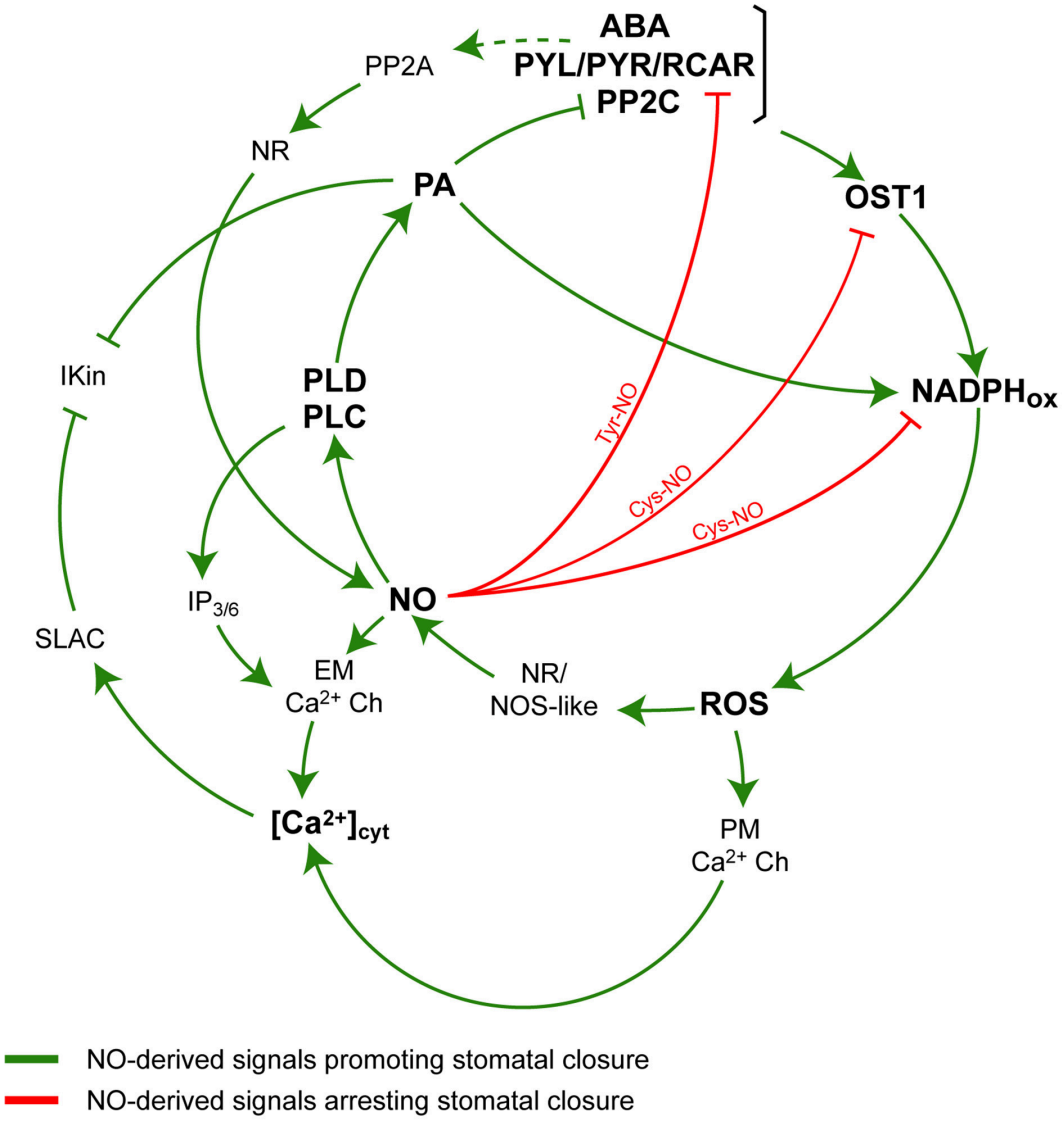
**A simplified model of ABA signaling in guard cells**. Nitric oxide (NO) promotes and attenuates the ABA-induced and phospholipid-mediated stomatal closure. The positive ABA-stimulus inducing the stomatal closure and involving NO and phospholipid-derived signals are in green. The negative effects of NO linked to post-translational modifications of proteins and attenuating the ABA signaling are in red. The model shows that ABA binds to its receptor pyrabactin resistance/regulatory component and recruits the protein phosphatase 2C [ABA-PYL/PYR/RCAR-PP2C], resulting in the activation of the kinase open stomata 1 (OST1). Then, OST1 phosphorylates and activates NADPH oxidase (NADPHox), with the consequent generation of reactive oxygen species (ROS) and downstream, the formation of NO through the enzymatic activities nitrate reductase (NR) and NO synthase-like (NOS-like). NO induces the formation of phosphatidic acid (PA) via the activation of phospholipase C (PLC) and phospholipase D (PLD) by a still unknown mechanism. PA in turn activates NADPH_ox_ and inhibits PP2C and inward-rectifying K^+^ (I_K, in_) channels. The activity of PLC also generates inositol phosphates (IP_3∕6_) contributing to the release of Ca^2+^ from intracellular stores through endomembranes Ca^2+^-channels (EM Ca^2+^ Ch). The increase of cytosolic Ca^2+^ concentration ([Ca^2+^]_cyt_) activates slow -anion channels (SLAC) which also inhibits I_K, in_. The production of ROS also participates in the regulation of [Ca^2+^]_cyt_ through the activation of plasma membrane Ca^2+^ channels (PM Ca^2+^ Ch). The model also shows a pathway proposing that ABA is able to induce the production of NO via the dephosphorylation and activation of NR through the activity of protein phosphatase 2A (PP2A) (Heidari et al., 2011). The attenuating effects of NO by breaking the ABA stimulus include the inhibition and degradation of the ABA receptor PYL/PYR/RCAR through the nitration of Tyr residues (Tyr-NO), and the inactivation of OST1 and NADPH_ox_ via S-nitrosylation (S-NO).

The production of the second messenger nitric oxide (NO) is required for ABA-dependent induction of stomatal closure (Desikan et al., 2002; Garcia-Mata and Lamattina, 2002; Neill et al., 2002; Suhita et al., 2004; He et al., 2005; Kolla et al., 2007). NO regulates a subset of ABA-evoked responses by inactivating I_K, in_ channels via a cGMP/cADPR-dependent increase of cytosolic Ca^2+^ concentration ([Ca^2+^]_cyt_) (Garcia-Mata et al., 2003). NO also induces the production of the lipid second messenger phosphatidic acid (PA) in guard cells (Distefano et al., 2008). PA is generated by phospholipase D (PLD) or by PLC through the hydrolysis of polyphosphoinositides (PPIs) in concerted action with diacylglycerol kinases. In addition, the hydrolysis of PPIs by PLCs also produces water-soluble inositol polyphosphates (InsPPs), that diffuses to the cytosol, promoting the release of Ca^2+^ from intracellular stores in guard cells (Lemtiri-Chlieh et al., 2003) and contributing to the increase of [Ca^2+^]_cyt_. Results have shown that NO-induction of stomatal closure was impaired when either PLC or PLD activity was inhibited (Distefano et al., 2008). These evidences suggest that PLD and PLC are participating in the NO-signaling pathway in guard cells (Distéfano et al., 2010). Regarding PA, it binds to both RbohD and RbohF, increasing their activity and leading to superoxide (O${}_{2}^{.-}$) production and H_2_O_2_ formation, and thereby contributing to the induction of stomatal closure (Zhang et al., 2009). In addition, it has been shown that PA interacts with and inhibits ABI1 (PP2C) (Zhang et al., 2004), and activates SnRK2s type I SnRK2.4 and 2.10 (Testerink et al., 2004) and PP2A (Gao et al., 2013), all of them components of the ABA signaling. Yet, there is no conclusive evidence supporting that both NO and PA production is via the activation of the PYL/PYR/RCAR receptor. Figure 1 summarizes the core of the signaling components under the control of NO and PA downstream ABA that, once integrated, determine the control of stomatal movements. There, it is highlighted the dual and compensatory mechanisms exerted by NO in the promotion and attenuation of the ABA-stimulated stomatal closure.

## Breaking the sense of the impulse, the no-mediated attenuation of ABA signaling in guard cells

One of the most intriguing and less understood processes in signal transduction is how do cells put a brake to multi-directional signal cascades with just one output. New available evidences suggest that NO could also function as blocker of the ABA-induced stomatal closure through the inhibition of the signaling by post-translational modifications of some key components of the cascade. The S-nitrosylation of Cysteine residues by NO-derived compounds is considered the most important NO-dependent post-translational modification of proteins due to its versatility and occurrence under physiological conditions (Astier and Lindermayr, 2012). It was demonstrated that Arabidopsis RbohD ability to form ROS is negatively regulated by the *S*-nitrosylation in cell death processes and immunity (Yun et al., 2011). The S-nitrosylation of Cys 890 of the Arabidopsis RbohD was sufficient to abolish its activity of forming ROS intermediates and consistently, its mutation also blocks any possibility of regulating NADPHox enzymatic activity. Moreover, Cys890 is conserved and also S-nitrosylated in humans and fly, suggesting a conserved post-translational regulatory pathway of NADPHox during evolution (Yun et al., 2011). As stated above PA binds to RbohD, and the PA-binding motif localizes in amino acid residues 101–330 (Zhang et al., 2009). In this region, mutation of the arginine residues 149, 150, 156, and 157 in RbohD resulted in the loss of PA binding and the loss of the activation of RbohD by PA (Zhang et al., 2009). It would be interesting to know if there exists any structural interference between the binding of PA and the S-nitrosylation of RbohD.

In a general view of the regulating process governing ABA-induced stomatal movement, NO could first induce lipid and lipid-derived molecules which activate NADPHox, but at a later time point, and probably based on increased and damaging concentrations of H_2_O_2_ and NO, NO is able to stop ROS production by inhibiting NADPHox activity directly by S-nitrosylation (Yun et al., 2011). Nevertheless, the NO-dependent post-translational modifications on RbohD still need to be proven in guard cells.

As stated, OST1 is a serine/threonine protein kinase that acts as a positive regulator mediating the ABA-induced stomatal closure through the activation of downstream effectors (Wang et al., 2013). In a very nice piece of work, two years later, Wang et al. (2015) demonstrated that NO negatively regulates ABA signaling in guard cells through the S-nitrosylation of OST1. NO can S-nitrosylates OST1 *in vitro* and *in vivo* at cysteine 137, a residue adjacent to the kinase catalytic site, provoking the dysfunction of its phosphorilating activity (Wang et al., 2015). At a first glance, it can be perceived that NO possesses a multitasking capacity of modulating ABA signaling in guard cells through a complex biological activity. It includes both positive and negative effects that can be summarized as an attenuated mechanism for the regulation of stomatal closure induced by ABA, in a smooth and continuously highly controlled adjustment. Figure 1 details the interactions occurring in guard cells highlighting the positive and negative effects of NO on the phospholipid-derived signals and the ABA-induced signaling resulting in stomatal closure. It includes (A) direct positive effects (increase of [Ca^2+^]_cyt_ and PA) and (B) negative effects leading to the attenuation of the ABA signaling through the inhibition of key effectors of stomatal closure (inhibition of NADPHox and OST1 by S-nitrosylation). A recently published article adds new *in vitro* and *in vivo* evidences showing that the family of ABA receptors PYR/PYL/RCAR is inactivated by nitration of tyrosine residues leading to the degradation of the receptor via proteasome. The non-reversible nitration of tyrosine residues is a post-translational modification of proteins that requires the formation of the strong oxidant peroxynitrite, a compound formed from the fast reaction between superoxide (O${}_{2}^{.-}$) and NO. In addition, the article shows that the ABA receptor is also S-nitrosylated, resulting in a full capacity of the receptor of inhibiting PP2C activity (Castillo et al., 2015). Even if authors speculate about the relevance of the S-nitrosylation and Tyr-nitration as a NO-mediated mechanism that modulates the ABA receptor biological activity, it was not yet proved whether it is functionally active in guard cells under physiological conditions associated to drought stress. It would be interesting to see if an increase of ABA concentration after perceiving the drought stress is enough to promote the nitration and degradation of the ABA receptor, leading to the loss of the response to ABA and to the brake of ABA-induced stomatal closure.

Overall, this opinion article tries to recall the already known two sides of the NO “coin” as a ubiquitous, homeostatic, and synchronizer molecule in cell physiology. Thereby, we highlight here the rationale of NO acting both in promoting and arresting the ABA-induced/phospholipid-mediated signals triggering the stomatal closure, as a way to avoid the exacerbation of a hormonal stimulus. In future investigations, however, it remains to be deciphered if the multi targets of NO are reached simultaneously or through a temporal and spatial pattern of its actions.

## Author contributions

The analysis, revision of the bibliography and the discussion of the data were conducted by AL, CG, and LL. The manuscript was prepared and written, including round of corrections, by AL, CG, and LL. The design and general supervision was performed by LL.

## Conflict of interest statement

The authors declare that the research was conducted in the absence of any commercial or financial relationships that could be construed as a potential conflict of interest.

## References

[B1] Astier J. Lindermayr C. (2012). Nitric oxide-dependent posttranslational modification in plants: an update. Int. J. Mol. Sci. 13, 15193–15208. 10.3390/ijms131115193 23203119 PMC3509635

[B2] Castillo M. C. Lozano-Juste J. Gonzalez-Guzman M. Rodriguez L. Rodriguez P. L. Leon J. (2015). Inactivation of PYR/PYL/RCAR ABA receptors by tyrosine nitration may enable rapid inhibition of ABA signaling by nitric oxide in plants. Sci. Signal. 8:ra89. 10.1126/scisignal.aaa7981 26329583

[B3] Desikan R. Graffiths R. Hancock J. Neill S. (2002). A new role for an old enzyme: nitrate reductase-mediated nitric oxide generation is required for abscisic acid-induced stomatal closure in Arabidopsis thaliana. Proc. Natl. Acad. Sci. U.S.A. 99, 16314–16318. 10.1073/pnas.252461999 12446847 PMC138608

[B4] Distefano A. M. Garcia-Mata C. Lamattina L. Laxalt A. M. (2008). Nitric oxide-induced phosphatidic acid accumulation: a role for phospholipases C and D in stomatal closure. Plant Cell Environ. 31, 187–194. 10.1111/j.1365-3040.2007.01756.x 17996010

[B5] Distéfano A. Lanteri M. ten Have A. García-Mata C. Lamattina L. Laxalt A. (2010). Nitric oxide and phosphatidic acid signaling in plants, in Lipid Signaling in Plants, Plant Cell Monographs, ed Teun M. (Berlin/Heidelberg: Springer), 223–242.

[B6] Gao H. B. Chu Y. J. Xue H. W. (2013). Phosphatidic acid (PA) binds PP2AA1 to regulate PP2A activity and PIN1 polar localization. Mol. Plant 6, 1692–1702. 10.1093/mp/sst076 23686948

[B7] Garcia-Mata C. Gay R. Sokolovski S. Hills A. Lamattina L. Blatt M. R. (2003). Nitric oxide regulates K^+^ and Cl^−^ channels in guard cells through a subset of abscisic acid-evoked signaling pathways. Proc. Natl. Acad. Sci. U.S.A. 100, 11116–11121. 10.1073/pnas.1434381100 12949257 PMC196936

[B8] Garcia-Mata C. Lamattina L. (2002). Nitric oxide and abscisic acid cross talk in guard cells. Plant Physiol. 128, 790–792. 10.1104/pp.011020 11891235 PMC1540215

[B9] He J. Xu H. She X.-P. Song X.-G. Zhao W.-M. (2005). The role and the interrelationship of hydrogen peroxide and nitric oxide in the UV-B-induced stomatal closure in broad bean. Funct. Plant Biol. 32, 237–247. 10.1071/FP04185 32689127

[B10] Heidari B. Matre P. Nemie-Feyissa D. Meyer C. Rognli O. A. Moller S. G.. (2011). Protein phosphatase 2A B55 and A regulatory subunits interact with nitrate reductase and are essential for nitrate reductase activation. Plant Physiol. 156, 165–172. 10.1104/pp.111.172734 21436382 PMC3091043

[B11] Kolla V. A. Vavasseur A. Raghavendra A. S. (2007). Hydrogen peroxide production is an early event during bicarbonate induced stomatal closure in abaxial epidermis of Arabidopsis. Planta 225, 1421–1429. 10.1007/s00425-006-0450-6 17160388

[B12] Kulik A. Wawer I. Krzywinska E. Bucholc M. Dobrowolska G. (2011). SnRK2 protein kinases–key regulators of plant response to abiotic stresses. OMICS 15, 859–872. 10.1089/omi.2011.0091 22136638 PMC3241737

[B13] Kwak J. M. Moon J. H. Murata Y. Kuchitsu K. Leonhardt N. DeLong A.. (2002). Disruption of a guard cell-expressed protein phosphatase 2A regulatory subunit, RCN1, confers abscisic acid insensitivity in Arabidopsis. Plant Cell 14, 2849–2861. 10.1105/tpc.003335 12417706 PMC152732

[B14] Lemtiri-Chlieh F. MacRobbie E. A. C. Webb A. A. R. Manison N. F. Brownlee C. Skepper J. N.. (2003). Inositol hexakisphosphate mobilizes an endomembrane store of calcium in guard cells. Proc. Natl. Acad. Sci. U.S.A. 100, 10091. 10.1073/pnas.1133289100 12913129 PMC187775

[B15] Ma Y. Szostkiewicz I. Korte A. Moes D. Yang Y. Christmann A.. (2009). Regulators of PP2C phosphatase activity function as abscisic acid sensors. Science 324, 1064–1068. 10.1126/science.1172408 19407143

[B16] MacRobbie E. (2006). Control of volume and turgor in stomatal guard cells. J. Membr. Biol. 210, 131. 10.1007/s00232-005-0851-7 16868673

[B17] Neill S. J. Desikan R. Clarke A. Hancock J. T. (2002). Nitric oxide is a novel component of abscisic acid signaling in stomatal guard cells. Plant Physiol. 128, 13–16. 10.1104/pp.010707 11788747 PMC1540198

[B18] Park S.-Y. Fung P. Nishimura N. Jensen D. R. Fujii H. Zhao Y.. (2009). Abscisic acid inhibits type 2C protein phosphatases via the PYR/PYL family of START proteins. Science 324, 1068–1071. 10.1126/science.1173041 19407142 PMC2827199

[B19] Pernas M. Garcia-Casado G. Rojo E. Solano R. Sanchez-Serrano J. J. (2007). A protein phosphatase 2A catalytic subunit is a negative regulator of abscisic acid signalling. Plant J. 51, 763–778. 10.1111/j.1365-313X.2007.03179.x 17617176

[B20] Sirichandra C. Gu D. Hu H. C. Davanture M. Lee S. Djaoui M.. (2009). Phosphorylation of the Arabidopsis AtrbohF NADPH oxidase by OST1 protein kinase. FEBS Lett. 583, 2982–2986. 10.1016/j.febslet.2009.08.033 19716822

[B21] Suhita D. Raghavendra A. S. Kwak J. M. Vavasseur A. (2004). Cytoplasmic alkalization precedes reactive oxygen species production during methyl jasmonate- and abscisic acid-induced stomatal closure. Plant Physiol. 134, 1536–1545. 10.1104/pp.103.032250 15064385 PMC419829

[B22] Testerink C. Dekker H. L. Lim Z.-Y. Johns M. K. Holmes A. B. de Koster C. G.. (2004). Isolation and identification of phosphatidic acid targets from plants. Plant J. 39, 527–536. 10.1111/j.1365-313X.2004.02152.x 15272872

[B23] Umezawa T. Sugiyama N. Mizoguchi M. Hayashi S. Myouga F. Yamaguchi-Shinozaki K.. (2009). Type 2C protein phosphatases directly regulate abscisic acid-activated protein kinases in Arabidopsis. Proc. Natl. Acad. Sci. U.S.A. 106, 17588–17593. 10.1073/pnas.0907095106 19805022 PMC2754379

[B24] Vlad F. Rubio S. Rodrigues A. Sirichandra C. Belin C. Robert N.. (2009). Protein phosphatases 2C regulate the activation of the Snf1-related kinase OST1 by abscisic acid in *Arabidopsis*. Plant Cell 21, 3170–3184. 10.1105/tpc.109.069179 19855047 PMC2782292

[B25] Waadt R. Manalansan B. Rauniyar N. Munemasa S. Booker M. A. Brandt B.. (2015). Identification of open stomata1-interacting proteins reveals interactions with sucrose non-fermenting1-related protein Kinases2 and with Type 2A protein phosphatases that function in abscisic acid responses. Plant Physiol. 169, 760–779. 10.1104/pp.15.00575 26175513 PMC4577397

[B26] Wang M. Yuan F. Hao H. Zhang Y. Zhao H. Guo A.. (2013). BolOST1, an ortholog of Open Stomata 1 with alternative splicing products in *Brassica oleracea*, positively modulates drought responses in plants. Biochem. Biophys. Res. Commun. 442, 214–220. 10.1016/j.bbrc.2013.11.032 24269232

[B27] Wang P. Du Y. Hou Y. J. Zhao Y. Hsu C. C. Yuan F.. (2015). Nitric oxide negatively regulates abscisic acid signaling in guard cells by S-nitrosylation of OST1. Proc. Natl. Acad. Sci. U.S.A. 112, 613–618. 10.1073/pnas.1423481112 25550508 PMC4299189

[B28] Yun B. W. Feechan A. Yin M. Saidi N. B. Le Bihan T. Yu M.. (2011). S-nitrosylation of NADPH oxidase regulates cell death in plant immunity. Nature 478, 264–268. 10.1038/nature10427 21964330

[B29] Zhang W. Qin C. Zhao J. Wang X. (2004). Phospholipase D alpha 1-derived phosphatidic acid interacts with ABI1 phosphatase 2C and regulates abscisic acid signaling. Proc. Natl. Acad. Sci. U.S.A. 101, 9508–9513. 10.1073/pnas.0402112101 15197253 PMC439007

[B30] Zhang Y. Zhu H. Zhang Q. Li M. Yan M. Wang R.. (2009). Phospholipase D(alpha)1 and phosphatidic acid regulate NADPH oxidase activity and production of reactive oxygen species in ABA-mediated stomatal closure in Arabidopsis. Plant Cell 21, 2357–2377. 10.1105/tpc.108.062992 19690149 PMC2751945

